# The Fate of Mitral Valve Surgery in the Pediatric Age: A 25-Year Single-Center Experience

**DOI:** 10.3390/jcm13133761

**Published:** 2024-06-27

**Authors:** Eitan Keizman, Shai Tejman-Yarden, Evyatar Hubara, Shay Illouz, Uriel Katz, David Mishaly, Alain E. Serraf, Uri Pollak

**Affiliations:** 1 Department of Cardiac Surgery, The Leviev Cardiothoracic and Vascular Center, Sheba Medical Center, Ramat-Gan 5266202, Israel; 2 Sackler School of Medicine, Tel Aviv University, Tel Aviv 6997801, Israel; 3 The Edmond J. Safra International Congenital Heart Center, Sheba Medical Center, Ramat-Gan 5266202, Israel; 4 Pediatric Critical Care Unit, Hadassah University Medical Center, Ein Kerem, Jerusalem 91240, Israel; 5 The Hebrew University Hadassah Medical School, Jerusalem 9112002, Israel

**Keywords:** mitral valve repair, mitral valve surgery, congenital mitral valve disease, acquired mitral valve disease

## Abstract

**Background:** The aim of this study was to evaluate the natural history of patients after mitral valve intervention in the pediatric age. **Methods:** This is a retrospective study including all patients who underwent mitral valve surgery from 1998 to 2022. The patients’ surgical reports, postoperative records, and ambulatory visits were reviewed. The endpoints of the study were survival and freedom from mitral valve reoperation. **Results:** Of the 70 patients included in the cohort, 61 patients (86.7%) had congenital mitral valve disease, of whom 46 patients (75.4%) had a predominantly mitral regurgitation lesion, and 15 patients (24.6%) had a predominantly mitral stenosis. In the mitral regurgitation group, all of the patients underwent valve repair with an operative mortality of one patient (2.1%), and with median follow-up of 4 years (range, 0.5–13 years), there was 4.3% mortality (*n* = 2) and 71.2% freedom from reoperation. In the mitral stenosis group, 11 patients underwent mitral valve repair, and 4 patients underwent valve replacement. There was an operative mortality of two patients (13.3%). With a 2-year median follow-up (range: 0.1–23 years), there were no additional mortality cases in the mitral stenosis group. All three patients who survived primary mitral valve replacement (100%) and four patients who survived a primary repair (40.0%) underwent reoperation. **Conclusions:** This study demonstrates encouraging outcomes for mitral valve repair. The mortality of patients with congenital mitral valve disease may also be related to a difficult postoperative course, rather than the MV lesion itself.

## 1. Introduction

In children, mitral valve (MV) diseases range from mitral clefts, which are commonly observed in patients with an atrioventricular canal (AVC), to complex deformities, which are often observed in congenital heart defects (CHD) that involve the left ventricle (LV).

In congenital MV disease, several components of the valvular and sub-valvular apparatus may be involved, thus requiring extensive procedures that may involve the LV. Therefore, unlike MV diseases in adults, structural MV diseases in the young, whether congenital or acquired, are somewhat challenging to classify and standardize, since their morphology and pathophysiology is unique to each patient [1]. Any component of the MV apparatus may be involved, resulting in valve stenosis, regurgitation, or both, which makes the surgical approach to MV diseases particularly challenging and the choice of valve reconstruction or valve replacement problematic [2,3].

In comparison to a mitral valve replacement (MVR), which is associated with significant morbidity and mortality, the retention of the native mitral valve offers some distinct advantages [4]. The repair of the MV apparatus preserves the geometry of the LV, the native valve does not require anticoagulation therapy [4], and it is associated with a lower risk of complications such as complete heart block, hemolysis, and endocarditis [5,6], all of which result in long-term survival and a better quality of life [7,8,9,10].

Since MV diseases are rare, experience with MV interventions is relatively limited and may differ from one institution to another. We reviewed our 25-year single-center experience to evaluate the natural history and management strategies of these complex patients. Specifically, the purpose of this study was to assess the mortality and freedom from reoperation for all pediatric patients who had surgical mitral valve disease intervention.

## 2. Materials and Methods

### 2.1. Study Design

This retrospective observational study was conducted at the Edmond J. Safra International Congenital Heart Center. It was approved by the Sheba Medical Center internal review board. From January 1998 to December 2022, a total of 118 patients underwent MV surgical intervention. The patients who underwent MV intervention after a previous AVC defect repair were excluded from the cohort. Other exclusion criteria included patients with single ventricular morphology, due to their distinctive anatomy and physiology, and those with no recorded follow-up (*n* = 10). Patients with Shone’s complex who were destined to receive a biventricular repair were included in the cohort. The criterium for a biventricular repair was a well-functioning left ventricle in an adequate size diagnosed in several preoperative echocardiograms. The cohort included patients with CHD requiring surgical intervention who had acquired MV disease consisting of rheumatic heart disease (RHD) and MV infective endocarditis (IE). The patients were divided, according to their pathology, into the mitral regurgitation (MR) group, the mitral stenosis (MS) group, and a third group consisting of patients with acquired MV disease.

### 2.2. Surgical Strategy

All procedures were performed by surgeons experienced in MV surgery. The operations were performed either by median sternotomy or right anterolateral thoracotomy. The thoracotomy procedures were performed using femoral cannulation on a fibrillating heart. Preoperative, as well as operative and postoperative, transesophageal echocardiography (TEE) evaluations of the MV were performed in all cases. During the MS repair procedures, an appropriate size Hagar dilator was inserted through the MV to assess adequate passage of blood across the valve orifice. As a rule, MV repair was the surgeon’s primary objective. Attempted repair that resulted in more than moderate MR or MS on TEE was concluded in valve replacement.

### 2.3. Mitral Valve Assessment and Data Collection

The data were collected by reviewing the medical records, operative notes, and pediatric cardiologist ambulatory notes, including transthoracic echocardiography (TTE) findings performed pre- and postoperatively and the TEE findings during the operation. The follow-up consisted of periodical visits, including TTE and a pediatric cardiologist specialist visit. The patients requiring further evaluation, including advanced scans and/or cardiac catheterization, were further evaluated accordingly. The congenital MV pathologies were classified according to Carpentier pathophysiological classification [11]. The MV pathology was determined by a combination of the preoperative TTE and TEE findings and the operative notes.

The echocardiographic data included the mean gradient across the MV, measured using continuous wave Doppler. The LV shortening fraction (SF; LVEDd–LVESd/LVEDd) was obtained either from M-mode or 2D parasternal long axis images. The MR was graded as mild, moderate, moderate to severe, or severe, based on qualitative parameters of the color-flow jet area and quantitative measures such as the vena contracta width and regurgitant fraction [12]. The MS was graded on the basis of the mean pressure gradient across the valve as mild, <5 mmHg; moderate, 5–10 mmHg; and severe, >10 mmHg [13].

The primary endpoints of this study were hospital and long-term mortality and freedom from surgical reintervention. The secondary endpoints were postoperative complications. The postoperative complications were categorized as minor (pneumothorax, chylothorax, pneumonia, and transient arrhythmia) or major complications (revision for tamponade, cerebral vascular accident (CVA), permanent arrhythmia requiring pacemaker implantation, postoperative low cardiac output and extracorporeal membrane oxygenation (ECMO) support requirement, acute kidney injury requiring peritoneal dialysis, and sepsis).

### 2.4. Statistical Analysis

The data are presented as the mean and standard deviation. The continuous variables are expressed as the median and ranges. The categorical variables are given as frequencies and percentages. Mortality and freedom from reintervention were assessed with Kaplan–Meier (KM) plots and the log-rank test. Associations between possible confounders and overall survival were investigated using univariate Cox proportional hazard models. In light of the relatively small cohort, multivariate models were not considered. Statistical significance was assumed when the null hypothesis could be rejected at *p* < 0.05. All *p*-values were the results of two-tailed tests. The statistical analyses were conducted using R (version 3.4.1).

## 3. Results

### 3.1. Patients with Congenital MV Disease

#### 3.1.1. Overall

**Figure 1:**
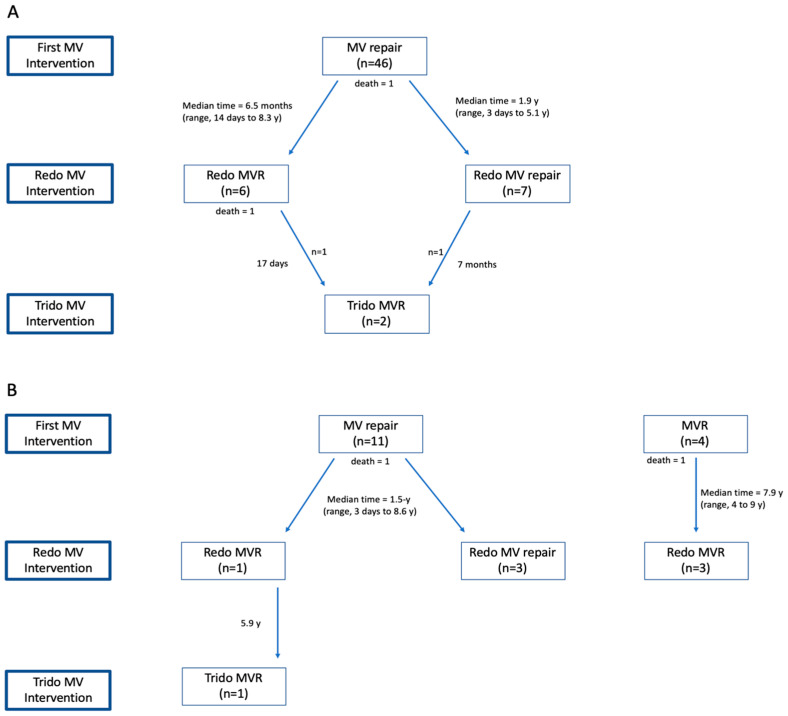
The clinical course for patients with congenital mitral valve disease. (**A**) presents patients with mitral regurgitation as their primary lesion. All of the patients in this group underwent mitral valve repair as the first surgical mitral valve intervention. (**B**) covers patients with mitral stenosis as their primary lesion. Four patients in this group underwent a mitral valve replacement, and eleven underwent a mitral valve repair. (MV—mitral valve; MVR—mitral valve replacement; y—years).

Of the 70 patients included in the cohort, 61 patients (86.7%) had congenital MV disease, and 9 patients (13.3%) had acquired MV disease. Out of the 61 patients with congenital MV disease, 46 (75.4%) had a predominantly MR lesion (MR group), and 15 (24.6%) had a predominantly MS lesion (MS group). The natural history of the patients with congenital MV disease appears in Figure 1.

#### 3.1.2. Mitral Regurgitation Group

Of the MR patients, 17 (36.2%) were males, with a median age of 24 months (range, 20 days to 10.5 years) and a weight of 11.4 kg (range, 2.6–34.5) (Table 1). Based on the Carpentier classification, the type I mitral pathology (normal leaflet motion) was most common, and present in 26 patients (56.5%). Type II lesions (leaflet prolapse) were present in 9 patients (19.5%), and 11 patients (23.9%) experienced type III lesions (restricted leaflet motion). A detailed description of the MV pathologies can be found in Table 2. In the MR group, 23 (50.0%) of the patients underwent posterior commissuroplasty, 21 (45.6%) cleft repair, and 8 (17.3%) patients underwent chord implantation. Different types of annuloplasties were used in 14 (30.4%) patients, including De Vega annuloplasty, Wooler–Kay annuloplasty, open-ring annuloplasty, and closed-ring annuloplasty (Table 3). The ring sizes ranged from 24 mm to 28 mm for the closed rings, and 16 mm to 26 mm for the open rings. Further details on the MR repair procedures appear in Table 3.

In the postoperative period, 14 patients (30.4%) experienced minor complications, and 2 patients (4.3%) suffered major complications, including low cardiac output in both patients, with 1 requiring ECMO support. In this case, the patient was weaned off ECMO after five days and was later discharged home.

Operative mortality included one patient (2.1%), an 18-month-old patient with a mitral cleft who, although having a relatively short cardiopulmonary bypass (CPB) time of 64 min, experienced low cardiac output in the immediate postoperative period and died on postoperative day one. One-year mortality was also found for one patient (2.1%) who presented with severe MR and aortic coarctation and underwent repair at the age of one month. They returned for redo MVR at the age of four months, and died postoperatively from low cardiac output. In the MR group, 14 (30.4%) were discharged with mild MR, 7 (15.2%) patients with moderate MR, and 1 (2.1%) patient with severe MR. Five (10.8%) patients were discharged with mild MS and one (2.1%) with moderate MS.

**Figure 2:**
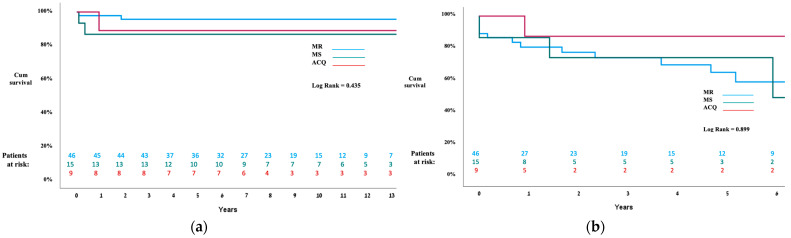
Kaplan–Meier curves for survival and freedom from mitral valve reoperation. (**a**) 13-year Kaplan–Meier curves for survival. There were no statistically significant group differences in the short- or long-term mortality. There was no long-term mortality. (**b**) 6-year Kaplan–Meier curves for mitral valve reintervention. No significant group differences were found, however, there were higher rates for mitral valve reintervention for the mitral stenosis group.

There were no additional mortality cases with a mean follow-up time of 4.3 ± 2.75 years and the median follow-up time of 4 years (range, 4 months to 13 years). The survival KM curve is presented in Figure 2.

In this group, 13 patients (28.8%) required reoperation, out of whom 7 patients (53.8%) underwent a redo MV repair and 6 patients (42.2%) had a redo MVR. The median time for a redo MV repair was 1.9 years (range, 3 days to 5.1 years), and the median time for a redo MVR was 6.5 months (range, 14 days to 8.3 years). Out of the seven patients undergoing a redo MV repair, only one patient underwent a third mitral intervention, which eventually concluded in an MVR. This was a female patient with a hypertrophic left ventricle and absent chords, who ended up with an orthotopic heart transplantation after four consecutive operations. Of the six patients undergoing a redo MVR, one patient died during the immediate postoperative period and one patient underwent transcatheter balloon expansion of the Melody valve. The KM curve for freedom from reintervention is presented in Figure 2a, and the natural history of the patients with MR is presented in Figure 1A.

#### 3.1.3. Mitral Stenosis Group

The MS group was composed of 15 patients, of whom 9 patients (60.0%) were male with a median age of 24 months (range, 11 days to 6 years) with a weight of 9.7 kg (range, 3.1–20 kg). According to the Carpentier classification of mitral pathology, eight patients (53.3%) presented with MS and a normal papillary muscle (Type A), and seven patients (46.6%) had an abnormal MV pathology (Type B). Shone’s Complex was diagnosed in four patients (26.6%). The other cardiac anomalies are listed in Table 1.

In all patients, the intent was MV repair; however, four patients (26.6%) ended up with an MVR. These procedures included one patient with Shone’s complex with a supravalvular membrane and a small valvular apparatus. The attempted repair in this patient consisted of papillary muscle splitting and a patch augmentation. Two patients underwent papillary muscle splitting alone for parachute mitral valve. A fourth patient had short chordae and underwent neochord implantation. In these cases, 16 mm to 23 mm mechanical valves were used. One patient underwent MVR with Carbomedix 19 mm at 1.5 years old, and, later, at the age of 9 years, received a 27 mm ATS valve. A second patient also had a Carbomedix 21 mm at the age of 2 years and also underwent a St. Jude 27 mm MVR at 12 years of age. This patient developed an atrioventricular block postoperatively, however, currently, at the age of 28 years, is doing well and practices sports. These two patients did not experience any thrombotic complications. The third patient received a St. Jude 19 mm valve at 4 months old and shortly after had a stuck valve. He underwent a redo MVR with Magna Ease 21 mm. The fourth and last patient among the patients who primarily underwent MVR had a 16 mm ATS MVR at a supra-annular position (unlike the other three who had an annular implantation). He unfortunately died postoperatively. This patient also had a smallish LV. The other 11 patients (73.3%) underwent various types of combined MV repair techniques, including papillary muscle splitting in 6 (54.5%), supravalvular ring resection in 6 (54.5%), commissurotomy in 4 (36.3%), and leaflet augmentation in 3 (27.2%). One patient (9.0%) had a 23 mm open ring annuloplasty. Further details on the MS repair techniques appear in Table 3.

During the immediate postoperative course, three patients (20.0%) experienced minor postoperative complications, and six patients (40.0%) had a major complication, including low cardiac output requiring ECMO support, CVA, acute kidney injury, and sepsis. Operative mortality was found for two patients (13.3%) including a 2.5-month-old male patient with Shone’s complex with a mildly hypoplastic LV and severe pulmonary hypertension. This patient underwent a prolonged CPB and cross-clamp time of 163 min and 139 min, respectively, eventually requiring an MVR. This patient died on day one postoperatively. The second operative mortality case involved a 3-month-old male with severe MS, aortic coarctation, and total anomalous pulmonic venous drainage (TAPVD). He too had a very prolonged CPB time of 306 min, with 54 min of cross-clamp time of his MV repair operation, which contributed to the poor outcome. Several hours after returning to the pediatric cardiac intensive care unit (PCICU), he required ECMO support. He eventually died on postoperative day 45. At the mean follow-up time of 5.6 (±6.7) years and the median follow-up time of 2 years (range, 1 month to 23 years), there were no additional mortality cases, as presented in the KM mortality curve (Figure 2). In the MS group, four (40.0%) out of the ten patients who underwent MV repair were discharged with mild MR and one (10.0%) patient with moderate MR. In terms of MS repair, three (30.0%) patients had mild MS and two (20.0%) had moderate MS at discharge.

In terms of reoperation for MS, all three patients who survived a primary MVR underwent a redo MVR with a median time for reintervention of 7.9 years (range, 4 to 9 years). One patient underwent a redo MVR due to a stuck valve, whereas the other two needed a valve replacement resulting from a patient–prosthesis mismatch. At a median time of 1.5 years (range, 3 days to 8.6 years), four patients (40.0%) who survived a primary MV repair underwent reoperation, three of whom had a redo repair procedure, and one had an MVR. This patient underwent a third mitral intervention (MVR) 5.9 years later. Altogether, seven patients (46.6%) in the MS group underwent an MV reoperation, as depicted in Figure 1B and in the KM curve for reintervention (Figure 2). The short- and long-term outcomes of the entire cohort are depicted in Table 4.

### 3.2. Patients with Acquired MV Disease

Nine patients presented with an acquired MV disease, of whom five patients (55.5%) were males. Seven patients had rheumatic heart disease (RHD) (77.7%), and two patients had bacterial IE (22.3%). All of the patients in this group presented with severe MR. The reconstruction techniques consisted of leaflet suture in one patient (11.1%), leaflet augmentation in two patients (22.2%), annuloplasty in four patients (44.4%), and chordal repair in three patients (33.3%).

There was no mortality or major complications in the immediate postoperative period. The median follow-up time of 2.7 years (range, 1 month to 9 years) showed that one patient underwent a reoperation 8 months after surgery and died postoperatively. This was a 15-year-old male patient with RHD involving the MV and the aortic valve for which he had undergone a redo triple valve surgery consisting of MVR, aortic valve replacement (AVR), and tricuspid valve (TV) repair. He developed sepsis and did not survive the postoperative course. The second redo case was a 16-year-old female patient with RHD who was operated on again for MV repair and TV repair 6.3 years after the initial intervention. Altogether, the long-term mitral reintervention rate was for two patients (22.2%).

## 4. Discussion

It is particularly challenging to manage congenital MV diseases, due to the complexity and variety of the different pathologies. Surgical reconstruction requires careful investigation and meticulous planning prior to surgery. Despite the different pathologies that cause MV dysfunction, there is a general consensus that valve reconstruction is the preferred option when treating MV in the pediatric population [13]. MV repair preserves the geometry of the LV, which may result in the preservation and even improvement of the ventricular function, thus improving the patients’ survival and morbidity [7]. A repaired valve does not require anticoagulation therapy [8,10,12] and is associated with a lower risk of complete heart block, hemolysis, and endocarditis compared to a prosthetic valve [7,8]. Previous studies have reported that MV repair may be successful in this age group, with long-term survival rates exceeding 79% [14,15]. Nevertheless, since most institutions have limited experience with MV surgery, there have been calls for a more comprehensive understanding of the outcomes in different mitral pathologies [8,14].

In this study, MV surgery for congenital MV disease demonstrated encouraging results of an overall 82.9% long-term survival rate, with lower in-hospital mortality in the MR group compared to the MS group (2.1% vs. 13.3%) (Figure 2). In a recent analysis, Mayr et al. found 79% 6-year survival in 40 patients with a similar median age, and, in another series by Geofftrion et al., 87.8% long-term survival was reported [12]. Interestingly, in this cohort, all of the patients who survived the postoperative course after MV surgery, regardless of pathology, with the exception of one case, were alive at the time of the latest follow-up (5.6 ± 6.7 years), including those who underwent MV reintervention. This finding suggests that mortality from this complex congenital anomaly may also be related to the difficult postoperative course rather than directly to the MV pathology. The explanations for a more complicated postoperative course among the MS group include the presence of left-sided obstructive defects such as Shone’s complex, which was more common in the MS group (26.6% vs. 4.3%), and the higher rate of concomitant procedures in the MS group (40.0% vs. 13.3%), which can prolong the cross-clamp time. However, despite experiencing more major postoperative complications (40.0% vs. 4.3%) and higher rates of in-hospital mortality (13.3% vs. 2.1%), the patients in the MS group had similar long-term survival rates as those in the MR group if they survived the immediate postoperative period, as presented in the KM curve (Figure 2).

In terms of freedom from reoperation, 46.6% of the patients in the MS group and 28.2% in the MR group required mitral valve reoperation (Figure 1). Though higher, the risk for MV reoperation in the MS group was not statistically significant. This finding is similar to a previously reported series of MV interventions [15,16,17,18]. Overall, the freedom from MV reintervention in this study was 67.3%. Similar rates of 61% and 67.7% were reported by Mayr et al. [15] and Chauvaud et al. [17], respectively, while better rates of 81.7% were described by Wood et al. [10].

It could be argued that the need for MV reintervention is not necessarily the result of an unsuccessful repair, but is rather due to the inevitable growth of the child, which is probably the major limitation to long-lasting valve reconstruction and the primary limitation of MVR success in the pediatric population. The results for MVR in the current study were discouraging. All of the patients who underwent primary MVR had an adverse outcome, with 25.0% mortality and a 75.0% reintervention rate at a median time of 7.9 years (range, 4 to 9 years). The four patients who underwent an MVR as a first intervention all presented with severe MS. One patient presented with Shone’s complex and died in the immediate postoperative period, one was a neonate with transposition of the great arteries (TGA), and the other two presented with papillary muscle abnormalities. MV repair was attempted in all four cases, but resulted in persistent significant MS or severe MR. A large retrospective study by Brancaccio et al. presented the results of 115 patients undergoing MVR. While their study included patients after a previous AVC repair, they demonstrated that the complication rate could be based on a small size/weight ratio [18]. The four patients in this cohort who underwent MVR also demonstrated a similar pattern. As previously described, two patients had 19 mm and 21 mm valves at a young age and survived decades later. A third patient with a 19 mm valve suffered a stuck valve, and the patient with a 16 mm valve died postoperatively. While no major conclusions can be deduced out of four cases, the largest prosthetic size should always be the goal of an MVR procedure.

When repairing MS, according to Poiseuille’s law, even limited relief in a stenotic valve allows the flow over the valve area to increase significantly. Thus, a slight alleviation of the degree of MS may dramatically improve the patient’s hemodynamics. Nonetheless, it is difficult to determine retrospectively whether settling for a less-than-perfect repair would have been a better option than replacing the valve in these four patients. Mitral valve balloon valvuloplasty has not been attempted in any of the patients with MS in this group. In light of some recent reports of the MV balloon valvuloplasty technique as a bridge for surgery, this technique should be considered whenever the valve pathology is favorable. This approach may delay the time of MVR and enable a larger prosthetic size [19].

Patients with acquired MV pathologies represent a different population than those with congenital MV disease. In this study, these patients were older, with a median age of 7.2 years (range, 2.1 to 16 years), and had no concomitant cardiac lesions. The outcomes in the acquired MV disease group were relatively good compared to the previously reported 65% to 79% long-term survival rates [20,21]. There was no postoperative mortality, and only one case of mortality within 1 year postoperatively. The overall long-term survival rate was 88.1%, with a median follow-up time of 2.7 years (range, 1 month to 9 years) and 78.8% freedom from reintervention. RHD is a disease that has dramatically decreased in prevalence in modern times, which explains the relatively low number of rheumatic patients undergoing MV interventions. Nevertheless, given the encouraging outcomes of these patients in this study, children with RHD may benefit from valve reconstruction, though no subgroup with MVR for comparison was present in this series.

### Limitations

This study has a number of limitations. First, it was a single-center, non-randomized retrospective trial subject to selection bias. Second, the patients were grouped into an MS or MR group as a function of their dominant lesion as stenosis or regurgitation, but some patients had coexisting MR and MS. Third, despite having congenital MV disease, these patients were relatively heterogeneous with diverse mitral pathologies, concomitant cardiac anomalies, and underwent different repair approaches. These differences may have contributed to confounds. Finally, the relatively small sample size limits the power and the ability to evaluate the effects of specific variables on outcomes.

## 5. Conclusions

The findings suggest encouraging outcomes for mitral valve repair in the pediatric age. Mitral valve replacement is usually reserved for particularly severe cases in which valve repair is unsuccessful. The morbidity and mortality of patients with congenital MV disease may also be related to a difficult postoperative course, regardless of the MV lesion. The results of MV replacement were discouraging.

## Figures and Tables

**Table 1:** The baseline patients’ characteristics, cardiovascular malformations and operative data of patients with congenital mitral regurgitation, congenital mitral stenosis, and acquired mitral valve disease.

	MR (*n* = 46)	MS (*n* = 14)	RHD/SBE (*n* = 9)
Median age, y (range, days–years)	2 (20–10.5)	2 (11–6)	7.2 (2.1 y–16 y)
Median weight, kg (range)	11.4 (2.6–34.5)	9.7 (3.1–20)	22.8 (10.3–60)
Median gestational age, weeks (range)	40 (30–41)	39 (33–42)	-
Median birth weight, gr (range)	3200 (843–4000)	3100 (1800–3600)	-
Male sex (%)	17 (36.9%)	9 (60.0%)	5 (55.5%)
Concomitant Cardiac Anomaly:			
VSD	12 (26.6%)	3 (20.0%)	-
ASD	5 (10.8%)	1 (6.6%)	-
Shone’s complex	2 (4.3%)	4 (26.6%)	-
ALCAPA	4 (8.6%)	-	-
ToF	1 (2.1%)	-	-
TGA	1 (2.1%)	1 (6.6%)	-
TAPVD	-	1 (6.6%)	-
PAPVD	1 (2.1%)	-	-
Ebstein’s Anomaly	1 (2.1%)	-	-
PLSVC	1 (2.1%)	-	-
Aortic Coarctation	-	2 (13.3%)	-
Aortic valve stenosis	-	1 (6.6%)	1 (11.1%)
>mod. LV Dysfunction	7 (15.2%)	1 (6.6%)	0%
Operative data *:			
Re-sternotomy	4 (8.8%)	5 (33.3%)	-
CPB time, min	81 (27–203)	89 (43–306)	88 (43–154)
XCP time, min	64 (22–177)	62 (32–179)	67 (35–123)
Temperature, °C	34 °C (27–36)	34 °C (20–36)	32 °C (30–36)
Concomitant Procedure	6 (13.0%)	6 (40.0%)	1 (11.1%)
Procedural ECMO	2 (4.3%)	0%	0%

Abbreviations: ALCAPA—anomalous left coronary artery origin from the pulmonary artery; ASD—atrial septal defect; CPB—cardiopulmonary bypass; ECMO—extracorporeal membrane oxygenation; LV—left ventricular; PAPVD—partial anomalous pulmonic venous drainage; TAPVD—total anomalous pulmonic venous drainage; TGA—transposition of the great arteries; ToF—tetralogy of Fallot; VSD—ventricular septal defect; XCP—cross-clamp; y—years. * presented as median and range.

**Table 2:** Mitral valve pathologies according to Carpentier’s classification of congenital mitral valve diseases.

Mitral Valve Insufficiency			46
**Type I** (normal leaflet motion)		26	
Cleft anterior leaflet	18		
Annular dilatation	7		
Leaflet defect	1		
**Type II** (leaflet prolapse)		9	
**Type III** (restricted leaflet motion)		11	
Type A (normal papillary muscle)	7		
Type B (abnormal papillary muscle)	4		
**Mitral Valve Stenosis**			**15**
**Type A** (normal papillary muscle)		8	
Supravalvular ring	6		
Leaflet fusion	2		
**Type B** (abnormal papillary muscle)		7	
Parachute	2		
Papillary muscle abnormality	5		

**Table 3:** A list of the mitral valve reconstruction techniques that were used in the primary mitral valve surgical intervention, divided according to techniques that were utilized for mitral valve regurgitation (46 cases) and those that were applied in cases of mitral valve stenosis (15 cases). Note that more than a single maneuver was applied for the same procedure.

Mitral Valve Insufficiency			
Cleft suture		21	45.6%
Posterior commissuroplasty		23	50.0%
Wooler–Kay annuloplasty		2	4.3%
De Vega annuloplasty		4	8.6%
Papillary muscle split		8	17.3%
Annuloplasty ring		8	17.3%
Open ring	6		
Closed ring	2		
Chords repair		8	17.3%
**Mitral Valve Stenosis**			
Papillary muscle split		6	54.5%
Repair of chordae		2	18.1%
Resection of supravalvular ring		6	54.5%
Commissurotomy		4	36.3%
Annuloplasty ring		1	9.0%
Open ring	1		
Leaflet augmentation		3	27.2%

**Table 4:** The immediate- and long-term outcomes of patients with congenital mitral regurgitation, congenital mitral stenosis, and acquired mitral valve diseases.

	MR (*n* = 46)	MS (*n* = 15)	RHD/SBE (*n* = 9)
Minor postoperative complications (PNX/AKI1-2/pneumonia/arrhythmia)	14 (30.4)%	3 (20.0%)	0%
Major postoperative complications (Revision/ECMO/AKI3/CVA/PPM)	2 (4.3)%	6 (40.0%)	0%
Ventilation days (range)	1 (0–37)	2 (0–45)	1 (0–4)
PCICU days (range)	3 (1–40)	4 (1–445	2 (1–6)
Hospitalization days (range)	8 (4–53)	12 (6–45)	8 (5–21)
In-hospital mortality	1 (2.1%)	2 (13.3%)	0%
1-year mortality	1(2.1%)	0%	1 (11.1%)
Long-term mortality	0%	0%	0%
Mitral reintervention	13 (28.8%)	4 (40%)	2 (22.2%)

Abbreviations: AKI—acute kidney injury; CVA—cerebrovascular accident; ECMO—extracorporeal membrane oxygenation; LV—left ventricular; PCICU—pediatric cardiac intensive care unit; PNX—pneumothorax; PPM—permanent pacemaker.

## Data Availability

Supporting data are available upon reasonable request.
